# Being an adult sibling of an individual with autism spectrum disorder may be a predictor of loneliness and depression – Preliminary findings from a cross-sectional study

**DOI:** 10.3389/fpsyg.2022.915915

**Published:** 2022-08-05

**Authors:** Kasper Sipowicz, Marlena Podlecka, Łukasz Mokros, Tadeusz Pietras, Kamila Łuczyńska

**Affiliations:** ^1^Department of Interdisciplinary Disability Studies, The Maria Grzegorzewska University in Warsaw, Warsaw, Poland; ^2^Department of Neuroses, Personality Disorders and Eating Disorders, Institute of Psychiatry and Neurology in Warsaw, Warsaw, Poland; ^3^Department of Clinical Pharmacology, Medical University of Lodz, Łódź, Poland; ^4^Second Department of Psychiatry, Institute of Psychiatry and Neurology in Warsaw, Warsaw, Poland; ^5^Department of Experimental Embryology, Institute of Genetics and Animal Biotechnology of the Polish Academy of Sciences, Jastrzębiec, Poland

**Keywords:** depression, loneliness, social support, autism, family

## Abstract

**Background:**

The aim of this study is to compare depression and loneliness among adult siblings of people on the autism spectrum, adult siblings of normotypic individuals, and adults raised alone (only child). In recent years, an increasing interest in the perspective of siblings of children diagnosed with autism has been observed, with studies among this population particularly concerned with the developmental trajectories of children and adolescents at “high risk” for ASD, rarely focusing on their mental well-being.

**Methods:**

The respondents filled out: the survey on sociodemographic data designed by the authors, Beck Depression Inventory II (BDI, measure of depression), and De Jong Gierveld Loneliness Scale (DJGLS, assessment of loneliness).

**Results:**

A rise in BDI and an increase in the DJGLS score were predicted by having a sibling diagnosed with ASD. Those effects were independent of subjects’ sex, educational status, place of residence, or a number of siblings.

**Conclusion:**

The results underline a fundamental need for the development of mental hygiene programs for families where children with autism spectrum are accompanied by healthy siblings.

## Introduction

The presence of an individual with autism spectrum disorder (ASD) is a powerful stressor for other members of the nuclear family (Bebko et al., 1987). Communication deficits in individuals with ASD disrupt communication across the whole family (Brignell et al., 2018). ASD behavioral disorders (aggression, self-stimulating movements, vocalizations, excessive motor activity, transgressing the interpersonal distance), sometimes persistent to the environment, can also impair significantly the functioning of the family as a whole (Gray and Holden, 1992). The existence of a child on the spectrum in the family affects the environment of healthy siblings’ development in a special way. In fact, as understood by the family system theory, the sibling subsystem is the first and strongest peer relationship, and thus its quality takes on particular importance in the further development of the young person (Angell et al., 2012). In a model describing the risk and resilience factors that affect the mental, behavioral and social functioning of siblings of children with ASD, in addition to the relationship between siblings, it is maternal functioning that also plays an important role in the formation of neurotypical siblings, and in particular indicators such as depression, the stress experienced or differentiated attention given to the children (Tudor et al., 2018).

The specific stressors that affect the psychosocial functioning of siblings of autistic individuals include changes in the roles of individual family members, reconstruction of functioning and activities undertaken by the family, loss or reduction of the parent’s attention, feelings of guilt, shame, embarrassment associated with the manifestation of specific behaviors by the autistic sibling (Randall and Parker, 1999). Social withdrawal, which can result in feelings of loneliness and isolation from other family members and peers, can be regarded as a typical response to such stressors (Benderix and Sivberg, 2007).

In recent years, a growing number of studies have been found to focus on family members of a child with autism, with the highest proportion of them concerning the psychosocial situation of parents and the degree of their adjustment to cope with the stressor, which is the child’s disability. An increasing interest in the perspective of siblings of children diagnosed with autism has also been observed, with studies among this population particularly concerned with the developmental trajectories of children and adolescents at “high risk” for ASD, rarely focusing on their mental well-being (Tudor et al., 2018). There have been no research studies addressing the issue of feelings of loneliness and covering the adult population of siblings of people with ASD.

It is often assumed *a priori* that having siblings diagnosed with ASD is associated with adverse experiences only, and thus with a negative impact on psychosocial functioning and a feeling of loneliness. However, the studies carried out to date fail to provide clear solutions in this regard. Some studies indicate that siblings of children with ASD experience more behavioral and emotional difficulties (Fisman et al., 1996; Hastings, 2003; Ross and Cuskelly, 2006; Meyer et al., 2011; Griffith et al., 2014). In contrast, other studies have not shown significant differences in the occurrence of abnormalities in these areas (Pilowsky et al., 2004; Dempsey et al., 2012). Other studies, in turn, demonstrate a greater level of adjustment of siblings of children with ASD compared to the general population (Hastings, 2003; Walton, 2016).

A study by Bagenholm and Gillberg carried out, among others, in a group of 20 children and young adults aged 5–20 who had a sister or a brother with autism demonstrated a high level of loneliness. As many as 35% of respondents admitted that they felt lonely, specifying it as a lack of friends, no contact with their peers, preferring to stay at home, and having to keep company with a neurodivergent brother or sister (Bågenholm and Gillberg, 1991). Kaminsky and Dewey, using the Loneliness and Social Dissatisfaction Questionnaire, found a low mean level of loneliness in that group, showing an additional negative correlation between the feeling of loneliness and mental adjustment (Kaminsky and Dewey, 2002).

There is a reported difference in the quality of the relationship between neurotypical siblings and individuals with intellectual disabilities often associated with autism as compared to the relationship between neurotypical siblings. There was less telephone contact and less expressed warmth as measured by the Five Minute Speech Sample in the first group (Doody et al., 2010) as well as less positive sibling relationship attitude as measured by Lifespan Sibling Relationship Scale alongside pronounced anxiety and depression symptomatology (Sommantico et al., 2020). Another study showcased higher levels of anxiety and depression symptoms in the siblings of individuals with autism, Prader-Willi syndrome, and developmental disability of unknown etiology (O’Neill and Murray, 2016). Higher levels of anxiety and depression among siblings of individuals with ASD were also noted in the metaanalysis along with worse functioning socially and psychologically (Shivers et al., 2019).

Some researchers have noted that neurotypical siblings of individuals with ASD who do not meet the ASD criteria themselves exhibit certain characteristics typical of autism. This phenomenon has been referred to as a broader autism phenotype (BAP) (Orsmond and Seltzer, 2007). A study by Lamport and Zlomke indicates that the presence of autistic traits of a severity known as the broader autism phenotype (BAP) is not a predictor of feelings of loneliness. However, as observed in a group of men, reduced levels of social skills and high social interaction anxiety are associated with a high level of loneliness (Lamport and Zlomke, 2014). In contrast, Jobe and White showed that subjects with stronger autistic phenotypes declared a significantly higher sense of loneliness while distinguishing themselves with fewer friendly relationships. However, in terms of the persistence of romantic relationships, a moderate positive correlation with the BAP indicator was noted. The researchers emphasize that BAP subjects do not prefer loneliness so much as they experience it because of their insufficient resources of social competencies (Jobe and Williams White, 2007). Looking at the results of the above studies, it should be borne in mind that the concept of BAP is not fully equivalent to the population of siblings of people with ASD.

In the area of mental well-being of adult siblings of ASD subjects, few studies which indicate the occurrence of certain emotional and social difficulties and a higher incidence rate of affective disorders in that group of people have been conducted so far (DeLong and Dwyer, 1988; Piven et al., 1990; Smalley et al., 1995; Orsmond and Seltzer, 2007). High severity of depressive symptoms and reduced social competencies are often associated with feelings of loneliness, understood as a discrepancy between the desired and currently available social relationship resources (Cacioppo et al., 2015; Cacioppo and Cacioppo, 2018).

Therefore, a question arises whether the adult siblings of people with the autism spectrum experience a stronger feeling of loneliness compared to those with normotypic siblings only. Does this feeling correlate with the severity of the depression and the selected demographic variables? The answer to this question can be fundamental for the development of mental hygiene programs for families where children with autism spectrum are accompanied by healthy siblings. The assessment of functioning during adulthood, after the end of adolescence, and the formation of the personality gives an answer concerning the extent to which the presence of autism in the family has a permanently destructive effect on the other siblings and promotes affective disorders.

The aim of this study is to compare depression and loneliness among adult siblings of people on the autism spectrum and adult siblings of normotypic individuals and adults raised alone (only child), in the context of selected sociodemographic variables.

## Materials and methods

### Study design and participants

The study was observational and cross-sectional in character. It was conducted from 2019 to 2021 among siblings of children with ASD treated at the Mental Health Outpatient Clinic in Aleksandrów Łódzki. The subjects without a sibling with ASD were recruited from among healthy volunteers who responded to an advertisement published by the researchers. Diagnosis of ASD was based on ICD-10 criteria.

Informed consent for participation in the study was a prerequisite for inclusion. The following exclusion criteria were applied: lack of informed consent, serious and unstable somatic disease, severe psychological trauma within 6 months preceding the study, and serious mental illness (e.g., schizophrenia, bipolar affective disorder, or another psychotic disorder).

The study procedure involved the completion of a set of self-reported questionnaires. The subjects were assessed regarding the exclusion criteria. After signing the informed consent, the participants were asked to complete a set of questionnaires: an authors’ survey on sociodemographic and clinical data and recognized psychometric tools (described below). Based on the completed questionnaires and clinical data, the patients were verified once again regarding the exclusion criteria. The study included 32 adults having a brother or a sister with ASD. Two control groups were included in the study. The first one consisted of 35 adults with a brother or sister without an ASD diagnosis. The second control group was composed of 34 people without any siblings. This second group was included as a control for the adults with neurotypical siblings. The selected demographic data of all groups are shown in Table 1.

**TABLE 1:** Comparison of the characteristics of the studied group of adults, who have a sibling with autism spectrum disorder (ASD), siblings without ASD and were the only child.

	Sibling with ASD (*N* = 32)	Sibling without ASD (*N* = 36)	Only child (*N* = 34)	Statistical test
Age, M ± SD (min-max)	31.1 ± 9.1 (18–48)	32.4 ± 9.7 (18–48)	31.0 ± 9.4 (18–46)	*F* = 0.801, df = 2, *p* = 0.802
**Sex, *N* (%)**				
Male	15 (48%)	15 (43%)	17 (50%)	*H* = 0.384, df = 2, *p* = 0.825
Female	16 (52%)	20 (57%)	17 (50%)	
**Level of education, *N* (%)**				
Secondary	14 (45%)	17 (49%)	20 (59%)	*H* = 1.456, df = 2, *p* = 0.483
Higher	14 (45%)	14 (40%)	12 (35%)	
Vocational	3 (10%)	4 (11%)	2 (6%)	
**Area of residence, *N* (%)**				
Rural	11 (35%)	13 (37%)	13 (38%)	*H* = 0.053, df = 2, *p* = 0.974
Municipal	20 (65%)	22 (63%)	21 (62%)	
**Number of siblings, *N* (%)**				
One	19 (61%)	18 (51%)	n/a	*U* = 475, *Z* = -0.971, *p* = 0.332
Two	10 (32%)	12 (34%)	n/a	
Three	2 (6%)	4 (11%)	n/a	
Four	0 (0%)	1 (3%)	n/a	
At least one younger sibling, *N* (%):	15 (48%)	16 (46%)	n/a	Chi2 = 0.047, df = 1, *p* = 0.828
At least one older sibling, *N* (%):	16 (52%)	19 (54%)	n/a	Chi2 = 1.397, df = 1, *p* = 0.237
BDI score, M ± SD (min–max)	9.8 ± 4.9 (4–23)	7.1 ± 3.3 (3–17)*	7.5 ± 3.5 (3–17)	*F* = 3.491, df = 2, *p* = 0.037
DJGLS score, M ± SD (min-max)	27.7 ± 14.0 (11–51)	18.9 ± 9.5 (11–41)*	24.8 ± 14.4 (11–55)	*F* = 4.992, df = 2, *p* = 0.010

*N*, number of observations; M, mean; SD, standard deviation; Min, minimum value; Max, maximum value; F, Welch test statistics; df, degrees of freedom; *H*, Kruskal–Wallis test statistics; Chi^2^, Pearson Chi-square statistics; *U* and *Z*, Mann Whitney *U* and *Z* test statistics; *p*, probability in the respective statistical test; BDI, Beck Depression Inventory II; DJGLS, De Jong Gierveld Loneliness Scale.

* *post hoc: p* < 0.05 vs. sibling with ASD group.

### Operationalization of the variables – Questionnaires

The Beck Depression Inventory version II (BDI) was used to assess the severity of depressive symptoms. The scale was adapted to Polish, validated, and issued by the Psychological Test Laboratory of the Polish Psychological Association. The test consists of 21 items concerning the occurrence and intensity of depressive symptoms within the past 2 weeks. Each item is scored from 0 to 3, which gives a total of 0 to 63 points. The higher the score, the greater the severity of depression (Beck et al., 1996; Zawadzki et al., 2009).

The assessment of loneliness was based on the De Jong Gierveld Loneliness Scale (DJGLS) in a Polish adaptation by Grygiel et al. (2013). The questionnaire comprises eleven items, five of which refer to the emotional dimension of loneliness, and six – to the social aspect. Each item is assessed based on a five-point answer scale. An increase in the DJGLS total score corresponds to a rise in the sense of loneliness (Grygiel et al., 2013).

The selected demographic details were collected in the form of a diagnostic survey – a questionnaire of the authors’ design, containing questions about age, sex, education level, place of residence, number of siblings, and the age of the siblings.

### Ethical considerations

The study was conducted in accordance with the institutional and national ethical standards and with the guidelines of the Declaration of Helsinki. Ethical review and approval were waived for this study, due to its observational character and the use of non-invasive measures (self-reported questionnaires).

### Statistical analysis

The STATISTICA 13.1 package with medical add-on software was used in the statistical analysis. The categorical variables were presented as a number with percentages. The associations between variables were assessed with Pearson’s Chi2 test in 2 × 2 contingencies. Mann–Whitney’s *U* test and Kruskal–Wallis *H* test were used for contingencies greater than 2 × 2. The continuous variables were characterized by their minimum-to-maximum range, mean value, and standard deviation. The normality of the distribution of the variables was verified with the Shapiro-Wilk test and visual analysis of the histograms. The central limit theorem was applied. Levene’s test was used to check the heterogeneity of variance between the subgroups. Intergroup comparisons were conducted by analysis of variance with Welch’s *t*-test, due to the lack of homogeneity of variance. In the case of intergroup differences that reached statistical significance, Tukey’s *post hoc* test was applied. Two linear regression models were constructed to predict the BDI score and DJGLS score. All variables of interest (characterized in Table 1) were regarded as potential predictors. The qualitative variables were coded with sigma restrictions. For each model, an analysis of residuals was performed to assess the validity of assumptions of normality, homoscedasticity, and independence between observations (with the Durbin–Watson test). To track possible multicollinearities, the tolerance indices were analyzed. The effect sizes were assessed in two manners: for each model as a whole (coefficient of determination *R*^2^) and each parameter in the model (semi- partial correlation sR). Those quotients may be interpreted in terms of Cohen’s thresholds for small (0.1), medium (0.3), and strong correlation (0.5). For both of the models, a 10-fold cross-validation was performed to assess their stability. The level of significance was adopted for α = 0.05. All variables of interest are characterized in Table 1.

## Results

### Intergroup comparisons

There was a statistically significant difference regarding the severity of loneliness and depression between the studied groups. The mean BDI and DJGLS scores were statistically significantly higher among individuals with ASD-diagnosed siblings compared to respondents with siblings without ASD, as shown in a *post hoc* analysis. The differences regarding the age, frequencies of sexes, area of residence, and level of education between the compared groups were not significant. The differences regarding the number of siblings and having an older or a younger sibling between the groups with an ASD sibling and siblings without ASD were not statistically significant (Table 1).

### Prediction of depression severity

The linear regression model constructed to predict the BDI score was adjusted to the empirical data (*R*^2^ = 0.350, *F* = 10.971, df = 12, *p* < 0.001). The above can be interpreted that the predictor variables that explained 35% of the variance in the BDI score.

There was a reduction in the cumulative correlation coefficient (and thus, the determination coefficient) for the model after ten-fold cross-validation: from *R* = 0.655 to *R* = 0.530. Yet, the difference between the coefficients was not statistically significant (*p* = 0.179), thus the model can be described as stable (Table 2).

**TABLE 2:** Parameters of the linear regression model predicting the Beck Depression Inventory score in the studied group of adults.

	B	B 95% CI		sR	*t*
Intercept	9.050	6.146	11.954		6.194
Age	–0.019	–0.097	0.059	–0.039	–0.486
Female sex	1.560	0.836	2.283	0.347	4.286
Area of residence: municipal vs. rural	1.150	0.314	1.986	0.221	2.733
Education: higher vs. secondary	–1.729	–2.908	–0.549	–0.236	–2.914
Vocational vs. secondary	2.606	0.847	4.364	0.239	2.946
Number of siblings: one vs. none	1.213	–0.763	3.188	0.099	1.220
Two vs. none	–0.434	–2.352	1.484	–0.036	–0.450
Three vs. none	–1.333	–4.350	1.684	–0.071	–0.878
Four vs. none	–0.534	–6.876	5.808	–0.014	–0.167
Sibling with ASD	1.425	0.562	2.288	0.266	3.282
At least one younger sibling	–0.393	–2.355	1.569	–0.032	–0.398
At least one older sibling	0.288	–1.653	2.229	0.024	0.294

B, unstandardized parameter; CI, confidence interval; sR, semipartial correlation (size of effect); *t*, statistics in *t*-test; *p*, probability in the *t*-test; ASD, autism spectrum disorder.

An increased BDI score was associated with female sex, living in a municipal area, having secondary vs. higher or vocational vs. secondary, and having a sibling with ASD (see Table 2 for detailed results of the regression analysis).

### Prediction of loneliness severity

The linear regression model constructed to predict the DJGLS score was adjusted to the empirical data (*R*^2^ = 0.366, *F* = 5.763, df = 12, *p* < 0.001). The above can be interpreted that the predictor variables that explained 37% of the variance in the DJGLS score.

There was a reduction in the cumulative correlation coefficient (and thus, the determination coefficient) for the model after ten-fold cross-validation: from *R* = 0.666 to *R* = 0.568. Yet, the difference between the coefficients was not statistically significant (*p* = 0.253), thus the model can be described as stable (Table 3).

**TABLE 3:** Parameters of the linear regression model predicting the De Jong Gierveld Loneliness Scale score in the studied group of adults.

	B	B 95% CI		sR	*T*
Intercept	28.639	19.456	37.821		6.199
Age	–0.144	–0.390	0.102	–0.093	–1.165
Female sex	4.692	2.405	6.979	0.326	4.078
Area of residence: municipal vs. rural	4.527	1.883	7.172	0.272	3.403
Education: higher vs. secondary	–3.036	–6.765	0.692	–0.130	–1.618
Vocational vs. secondary	5.445	–0.115	11.005	0.156	1.946
Number of siblings: one vs. none	1.837	–4.411	8.084	0.047	0.584
Two vs. none	–3.852	–9.917	2.213	–0.101	–1.262
Three vs. none	–5.970	–15.510	3.571	–0.100	–1.244
Four vs. none	–0.909	–20.963	19.145	–0.007	–0.090
Sibling with ASD	4.611	1.882	7.339	0.269	3.359
At least one younger sibling	0.373	–5.830	6.576	0.010	0.120
At least one older sibling	2.159	–3.978	8.297	0.056	0.699

B, unstandardized parameter; CI, confidence interval; sR, semipartial correlation (size of effect); *t*, statistics in *t*-test; *p*, probability in the *t*-test; ASD, autism spectrum disorder.

The female sex, living in a municipal area, and having a sibling diagnosed with ASD were the factors predictive of a rise in the DJGLS score (see Table 3 for detailed coefficients and effect sizes).

## Discussion

The levels of depression and loneliness were found to be higher in subjects who have siblings with ASD as compared with siblings of neurotypical individuals. This observation is in line with the literature data (Kaminsky and Dewey, 2002; Macks and Reeve, 2007). Feelings of loneliness and worsening depression in the group of people who have siblings with ASD can be explained by the negative effects of autism on the functioning of the entire nuclear family, including neurotypical siblings (Rao and Beidel, 2009). Autism is a powerful chronic stressor for parents, which undoubtedly has an important impact on the development of neurotypical siblings (Dabrowska and Pisula, 2010). The common genetic basis of autism and feelings of loneliness cannot be ruled out. Some neuropsychological deficits in healthy siblings of subjects with autism may promote feelings of loneliness and depression (Chen et al., 2016; Chien et al., 2017; Seng et al., 2020). Such deficits and differences in the white matter of the brain have been demonstrated in the siblings of people with autism as compared with those without autism in the family. In contrast, the severity of depression among only children was similar to that of neurotypical siblings, and the feeling of loneliness in that group was similar to that of siblings of children with autism spectrum. However, these differences were not statistically significant, which indicates a certain trend only. Having neurotypical siblings protects to some extent against feeling lonely (Cruise and O’Reilly, 2014; Jambon et al., 2019).

Interesting outcomes are also provided by qualitative research, which also indicates worse functioning of siblings of people from the autism spectrum than siblings of neurotypical people (Hodapp et al., 2010; Meadan et al., 2010; Atkin and Tozer, 2014; Mandleco and Webb, 2015). These studies are valuable in that they take into account the individual process of experiencing being a brother or sister of a neurodivergent person. Women in all studied groups felt lonelier than men and had greater severity of depression. This is in line with the well-known fact that unipolar affective disorders are two to three times more common in women than in men (Parker and Brotchie, 2010; Salk et al., 2017). An interesting observation in our study is the higher severity of depression and feelings of loneliness in people living in the city as related to people living in the countryside. Living in the city is associated with a weak social support network. Numerous professional contacts do not provide emotional support for an individual. In the rural areas, people are surrounded by members of the nuclear and extended families and have numerous relationships with neighbors and other villagers (Sipowicz et al., 2021). It has been confirmed unequivocally by the research that social network protects against depression. The interpersonal therapy of depression, which task is to build the social network of a person suffering from depression, is based on this fact (Cuijpers et al., 2011).

Our research also shows that a higher education level correlates with lower severity of depression and feelings of loneliness. Education is associated with cognitive and emotional resources, the launch of which in a crisis can prevent mood disorders and feelings of loneliness (Patel et al., 2019). This fact can also be looked at differently – people without depression and not feeling lonely are more likely to develop. Mental health is a prerequisite for the development of individuals and their transgression.

The limitation of our work is the lack of randomity in the study group selection. The results obtained for large populations may vary from the result obtained by us due to a relatively small size of our study group. Multicenter studies on a large population rather than based on two locations should be conducted, as the results may have been influenced by the specificity of the local community. The size of the samples also prevents us from analyzing birth order as we wouldn’*t* be able to reach sufficient statistical power.

Empirical studies have also demonstrated a number of variables not taken into account in this study to be risk factors for loneliness and depression. It would also be crucial to consider whether loneliness is the cause or the effect of depression, or whether both variables depend on common factors and affect each other in a circular way. However, those require further analysis, with proper considerations regarding the methodology, statistical models and tools to be applied, which would determine the nature and the cause-and-effect relationship.

## Conclusion

The levels of depression and loneliness were found to be higher in subjects who have siblings with ASD as compared with siblings of neurotypical individuals. In contrast, the severity of depression among only children was similar to that of neurotypical siblings, and the feeling of loneliness in that group was similar to that of siblings of children with autism spectrum. However, these differences were not statistically significant, which indicates a certain trend only. Women in all studied groups felt lonelier than men and had greater severity of depression. Our outcomes indicate an increased need for screening for depression and loneliness in siblings of individuals with ASD and may be of importance in the development of standards of care.

## Data availability statement

The original contributions presented in this study are included in the article/supplementary material, further inquiries can be directed to the corresponding author.

## Ethics statement

Ethical review and approval was not required for the study on human participants in accordance with the local legislation and institutional requirements. The patients/participants provided their written informed consent to participate in this study.

## Author contributions

KS, ŁM, and TP performed material preparation, data collection, and analysis. All authors wrote the first draft of the manuscript, contributed to the concept and design of the study, commented on previous versions of the manuscript, and read and approved the final manuscript.
